# Supplementary material for: Determining Cognitive Workload Using Physiological Measurements: Pupillometry and Heart-Rate Variability
Source: Sensors (Basel). 2024 Mar 21;24(6):2010. doi: 10.3390/s24062010 (PMC10974471; doi:10.3390/s24062010)
Supplement: Supplementary file 1 [file sensors-24-02010-s001.zip › sensors-2842448-supplementary.pdf]

Table S1. Non-parametric statistics results summary table for pupillometry parameter baselined pupil dilation

Statistic type	Variable	Complexity effect	Complexity: expertise effect
Wald-Type Statistic	Pupil dilation	Complexity: $\chi^2(3) = 77.664, P<0.001$***	$\chi^2(3)= 2.246, P=0.523$(ns)
ANOVA-Type Statistic	Pupil dilation	Complexity : $F(2.147) = 38.710, P < 0.001$***	$F(2.147)= 0.445, P= 0.655$(ns)

Table S2. Relative treatment effects summary table for pupillometry parameter baselined pupil dilation

Effect	Baseline(95% CI)	Low TC(95% CI)	Medium TC(95% CI)	High TC(95% CI)
Complexity	0.169	0.577	0.661	0.565
Complexity:expert	0.169(0.119, 0.245)	0.525 (0.382, 0.662)	0.652(0.489, 0.780)	0.534(0.379, 0.682)
Complexity:non-expert	0.169(0.124, 0.253)	0.630(0.515, 0.728)	0.671(0.538, 0.772)	0.596 (0.472, 0.704)

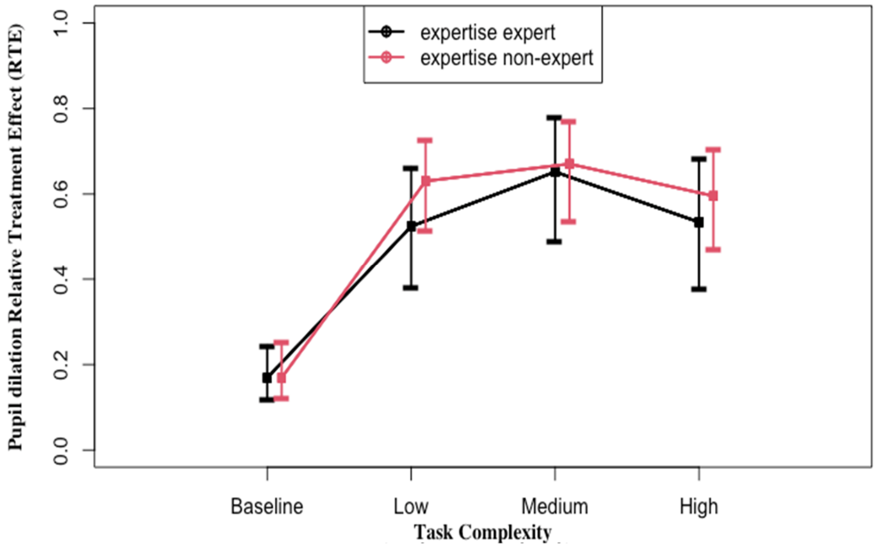

Figure S1. Relative treatment effects for pupillometry parameter baselined pupil dilation within the interaction of task complexity and expertise.

Table S3. Multiple comparisons of parameter baselined peak pupil dilation in the engine assembly study with Bonferroni adjustment.

**Comparison**	**Hypothesis**	**ANOVA-Type Statistic p-value**	**Adjusted p-value (Bonferroni adjustment)**
Baseline vs Low TC	Complexity:H0: $\mu_{baseline}=\mu_{low}$ Interaction:H0: $\mu_{experti}$ = $\mu_{non-experti}$ for i = baseline, low.	p-value(complexity)<0.0001*** p-value(interaction) = 0.584	p-value(complexity)<0.0001*** p-value(interaction) =3.504
Baseline vs Medium TC	Complexity:H0: $\mu_{baseline}=\mu_{medium}$ Interaction:H0: $\mu_{experti}$ = $\mu_{non-experti}$ for i = baseline,medium.	p-value(complexity)<0.001*** p-value(interaction)= 0.821	p-value(complexity)<0.001*** p-value(interaction)= 4.927
Baseline vs High TC	Complexity:H0: $\mu_{baseline}=\mu_{high}$ Interaction:H0: $\mu_{experti}$ = $\mu_{non-experti}$ for i = baseline,high.	p-value(complexity)= <0.001*** p-value(interaction) = 0.937	p-value(complexity)<0.001*** p-value(interaction)= 5.624
Low TC vs Medium TC	Complexity:H0: $\mu_{low}=\mu_{medium}$ Interaction:H0: $\mu_{experti}$ = $\mu_{non-experti}$ for i =low,medium.	p-value(complexity)= 0.026 p-value(interaction)= 0.268	p-value(complexity)= 0.154 p-value(interaction)= 1.609
Low TC vs High TC	Complexity:H0: $\mu_{low}=\mu_{high}$ Interaction:H0: $\mu_{experti}$ = $\mu_{non-experti}$ for i =low,high.	p-value(complexity)= 0.729 p-value(interaction)= 0.434	p-value(complexity)= 4.374 p-value(interaction)= 2.606
Medium vs High TC	complexity: H0: $\mu_{medium}=\mu_{high}$ Interaction:H0: $\mu_{experti}$ = $\mu_{non-experti}$ for i =medium,high.	p-value(complexity)= 0.024 p-value(interaction)= 0.527	p-value(complexity)= 0.146 p-value(interaction)= 3.161

Table S4. Non-parametric statistics results summary table for cardiac parameter baselined PNN50

**Statistic type**	**Variable**	**Complexity effect**	**Complexity:expertise effect**
Wald-Type Statistic	PNN50	$\chi^2(3)$ = 9.650,P=0.022*	$\chi^2(3)$= 10.074,P=0.018*
ANOVA-Type Statistic	PNN50	F(2.615) = 2.963, P=0.038*	F(2.615)= 2.273,P= 0.087(ns)

Table S5. Relative treatment effects summary table for cardiac parameter baselined PNN50.

**Effect**	**Baseline(95% CI)**	**Low TC(95% CI)**	**Medium TC(95% CI)**	**High TC(95% CI)**
Complexity	0.416	0.553	0.618	0.522
Complexity:expert	0.404(0.265, 0.566)	0.673(0.452, 0.828)	0.719(0.473, 0.866)	0.684 (0.498, 0.817)
Complexity:non-expert	0.427(0.306, 0.562)	0.434(0.311, 0.569)	0.516(0.337, 0.690)	0.361 (0.260, 0.485)

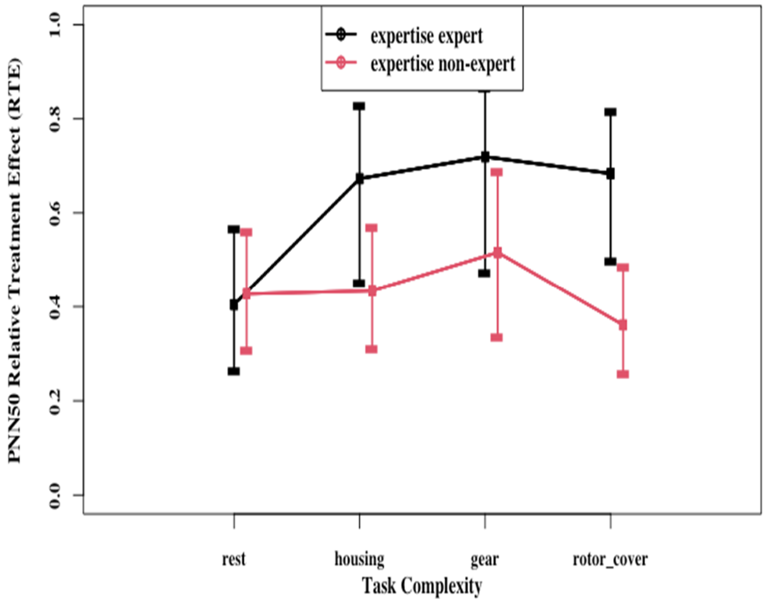

Figure S2. Relative treatment effects for cardiac parameter baselined PNN50 within the interaction of task complexity and expertise.

Table S6. Multiple comparisons of parameter baselined PNN50 in the engine assembly study with Bonferroni adjustment.

Comparison	Hypothesis	ANOVA-Type Statistic p-value	Adjusted p-value (Bonferroni adjustment)
Baseline vs Low TC	Complexity:H0: $\mu_{baseline}=\mu_{low}$ Interaction:H0: $\mu_{experti}$ = $\mu_{non\text{-}experti}$ for i = baseline, low.	p-value(complexity)= 0.022* p-value(interaction) =0.077	p-value(complexity)=0.135 p-value(interaction) =0.460
Baseline vs Medium TC	Complexity:H0: $\mu_{baseline}=\mu_{medium}$ Interaction:H0: $\mu_{experti}$ = $\mu_{non\text{-}experti}$ for i = baseline,medium.	p-value(complexity)= 0.006** p-value(interaction)= 0.046*	p-value(complexity)= 0.037* p-value(interaction)= 0.278
Baseline vs High TC	Complexity:H0: $\mu_{baseline}=\mu_{high}$ Interaction:H0: $\mu_{experti}$ = $\mu_{non\text{-}experti}$ for i = baseline,high.	p-value(complexity)= 0.195 p-value(interaction) =0.002**	p-value(complexity)= 1.172 p-value(interaction)= 0.015*
Low TC vs Medium TC	Complexity:H0: $\mu_{low}=\mu_{medium}$ Interaction:H0: $\mu_{experti}$ = $\mu_{non\text{-}experti}$ for i =low,medium.	p-value(complexity)= 0.157 p-value(interaction)= 0.674	p-value(complexity)= 0.941 p-value(interaction)= 4.043
Low TC vs High TC	Complexity:H0: $\mu_{low}=\mu_{high}$ Interaction:H0: $\mu_{experti}$ = $\mu_{non\text{-}experti}$ for i =low,high.	p-value(complexity)= 0.835 p-value(interaction)= 0.472	p-value(complexity)= 5.008 p-value(interaction)= 2.833
Medium vs High TC	complexity: H0: $\mu_{medium}=\mu_{high}$ Interaction:H0: $\mu_{experti}$ = $\mu_{non\text{-}experti}$ for i =medium,high.	p-value(complexity)= 0.037* p-value(interaction)= 0.376	p-value(complexity)= 0.224 p-value(interaction)= 2.255

Table S7. Non-parametric statistics results summary table for pupillometry cognitive workload index

Statistic type	Variable	Complexity effect	Complexity:expertise effect
Wald-Type Statistic	pupillometry index	Complexity: $\chi^2(3)$ =280.264,P<.001***	$\chi^2(3)$= 1.419,P=0.701(ns)
ANOVA-Type Statistic	pupillometry index	Complexity : F(2.413) =35.335, p < .001***	F(2.413)= 0.629, P=0.562(ns)

Table S8. Relative treatment effects summary table for pupillometry cognitive workload index

Effect	Baseline(95% CI)	Low TC(95% CI)	Medium TC (95% CI)	High TC(95% CI)
Complexity	0.196	0.503	0.447	0.819
Complexity:expert	0.191 (0.114, 0.325)	0.429 (0.303, 0.567)	0.445 (0.305,0.597)	0.777 (0.671, 0.850)
Complexity:non-expert	0.200 (0.150,0.276)	0.577 (0.443, 0.696)	0.449 (0.330, 0.578)	0.860 (0.800, 0.891)

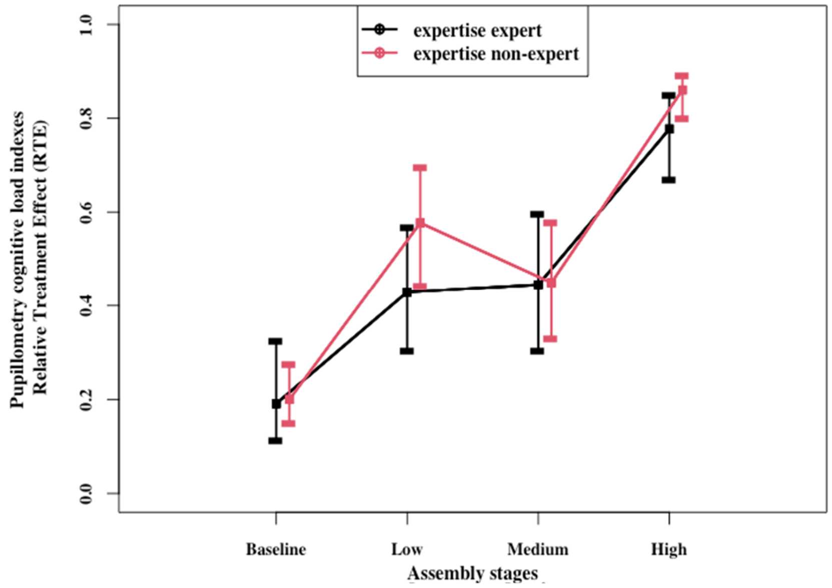

Figure S3. Relative treatment effects for pupillometry cognitive load index within the interaction of task complexity and expertise.

Table S9. Multiple comparisons of parameter pupillometry cognitive load index in the engine assembly study with Bonferroni adjustment

Comparison	Hypothesis	ANOVA-Type Statistic p-value	Adjusted p-value (Bonferroni adjustment)
Baseline vs Low TC	Complexity:H0: $\mu_{baseline}=\mu_{low}$ Interaction:H0: $\mu_{experti} = \mu_{non\text{-}experti}$ for i = baseline, low.	p-value(complexity)<0.0001**** p-value(interaction) = 0.514	p-value(complexity)<0.001*** p-value(interaction) =3.084
Baseline vs Medium TC	Complexity:H0: $\mu_{baseline}=\mu_{medium}$ Interaction:H0: $\mu_{experti} = \mu_{non\text{-}experti}$ for i = baseline,medium.	p-value(complexity)<0.0001**** p-value(interaction)= 0.820	p-value(complexity)<0.0001**** p-value(interaction)= 4.921
Baseline vs High TC	Complexity:H0: $\mu_{baseline}=\mu_{high}$ Interaction:H0: $\mu_{experti} = \mu_{non\text{-}experti}$ for i = baseline,high.	p-value(complexity)<0.0001**** p-value(interaction) = 0.331	p-value(complexity)<0.0001**** p-value(interaction)= 1.987
Low TC vs Medium TC	Complexity:H0: $\mu_{low}=\mu_{medium}$ Interaction:H0: $\mu_{experti} = \mu_{non\text{-}experti}$ for i =low,medium.	p-value(complexity)= 0.347 p-value(interaction)= 0.252	p-value(complexity)= 2.082 p-value(interaction)= 1.515
Low TC vs High TC	Complexity:H0: $\mu_{low}=\mu_{high}$ Interaction:H0: $\mu_{experti} = \mu_{non\text{-}experti}$ for i =low,high.	p-value(complexity)<0.0001**** p-value(interaction)= 0.758	p-value(complexity)<0.0001**** p-value(interaction)= 4.546
Medium vs High TC	complexity: H0: $\mu_{medium}=\mu_{high}$ Interaction:H0: $\mu_{experti} = \mu_{non\text{-}experti}$ for i =medium,high.	p-value(complexity)<0.0001**** p-value(interaction)= 0.407	p-value(complexity)<0.0001**** p-value(interaction)= 2.444

Table S10. Non-parametric statistics results summary table for cardiac cognitive workload index

**Statistic type**	**Variable**	**Complexity effect**	**Complexity:expertise effect**
Wald-Type Statistic	cardiac cognitive workload index	Complexity:$\chi^2(3)$ = 31.645,P<.001***	$\chi^2(3)$= 4.963,P=0.175(ns)
ANOVA-Type Statistic	cardiac cognitive workload index	Complexity: F(2.507) = 9.555, p <.001 ***	F(2.507)= 1.288, P= 0.277

Table S11. Relative treatment effects summary table for cardiac cognitive workload index.

**Effect**	**Baseline(95% CI)**	**Low TC(95% CI)**	**Medium TC (95% CI)**	**High TC(95% CI)**
Complexity	0.481	0.413	0.311	0.738
Complexity:expert	0.495(0.356,0.635)	0.279 (0.158,0.463)	0.306 (0.167, 0.510)	0.673(0.424,0.841)
Complexity:non-expert	0.467 (0.367,0.572)	0.548(0.407,0.678)	0.316(0.198,0.484)	0.804 (0.645, 0.878)

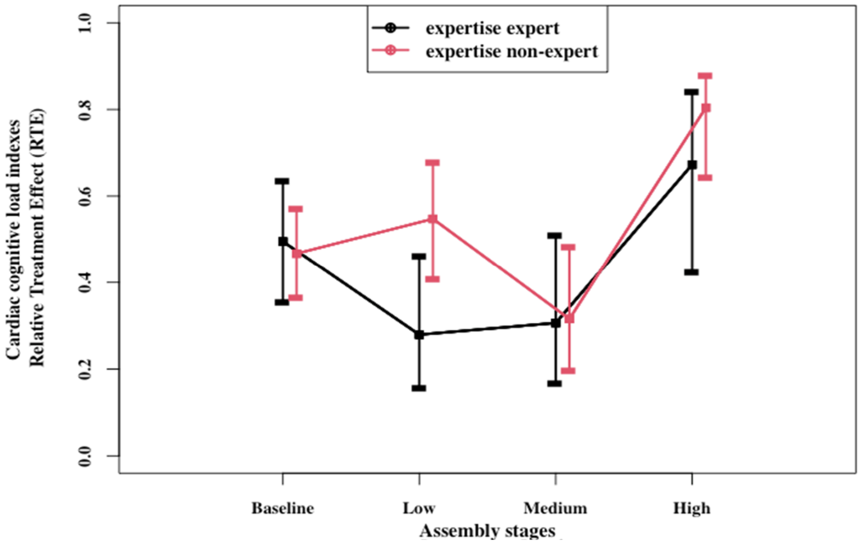

Figure S4. Relative treatment effects for cardiac cognitive load index within the interaction of task complexity and expertise.

Table S12. Multiple comparisons of parameter cardiac cognitive load index in the engine assembly study with Bonferroni adjustment

**Comparison**	**Hypothesis**	**ANOVA-Type Statistic p-value**	**Adjusted p-value (Bonferroni adjustment)**
Baseline vs Low TC	Complexity:H0: $\mu_{baseline}=\mu_{low}$ Interaction:H0: $\mu_{experti}$ = $\mu_{non\text{-}experti}$ for i = baseline, low.	p-value(complexity)= 0.244 p-value(interaction) = 0.038*	p-value(complexity)= 1.465 p-value(interaction) =0.231
Baseline vs Medium TC	Complexity:H0: $\mu_{baseline}=\mu_{medium}$ Interaction:H0: $\mu_{experti}$ = $\mu_{non\text{-}experti}$ for i = baseline,medium.	p-value(complexity)= 0.005** p-value(interaction)= 0.876	p-value(complexity)= 0.030* p-value(interaction)= 5.256
Baseline vs High TC	Complexity:H0: $\mu_{baseline}=\mu_{high}$ Interaction:H0: $\mu_{experti}$ = $\mu_{non\text{-}experti}$ for i = baseline,high.	p-value(complexity)<0.001*** p-value(interaction) = 0.349	p-value(complexity)<0.01** p-value(interaction)= 2.092
Low TC vs Medium TC	Complexity:H0: $\mu_{low}=\mu_{medium}$ Interaction:H0: $\mu_{experti}$ = $\mu_{non\text{-}experti}$ for i =low,medium.	p-value(complexity)= 0.247 p-value(interaction)= 0.174	p-value(complexity)= 1.482 p-value(interaction)= 1.047
Low TC vs High TC	Complexity:H0: $\mu_{low}=\mu_{high}$ Interaction:H0: $\mu_{experti}$ = $\mu_{non\text{-}experti}$ for i =low,high.	p-value(complexity)<0.001*** p-value(interaction)= 0.660	p-value(complexity)<0.01** p-value(interaction)= 3.959
Medium vs High TC	Complexity: H0: $\mu_{medium}=\mu_{high}$ Interaction:H0: $\mu_{experti}$ = $\mu_{non\text{-}experti}$ for i =medium,high.	p-value(complexity)<0.0001**** p-value(interaction)= 0.282	p-value(complexity)<0.0001**** p-value(interaction)= 1.690

Table S13. Non-parametric statistics results summary table for completion time

**Statistic type**	**Variable**	**Complexity effect**	**Complexity:expertise effect**
Wald-Type Statistic	Completion time	$\chi^2(3)$ = 552.035, P<.001***	$\chi^2(3)$= 3.523,P=0.318(ns)
ANOVA-Type Statistic	Completion time	F(2.119) = 68.986, P<.001***	F(2.119)= 0.717,P= 0.496(ns)

Table S14. Relative treatment effects summary table for variable completion time

**Effect**	**Baseline(95% CI)**	**Low TC(95% CI)**	**Medium TC(95% CI)**	**High TC(95% CI)**
Complexity	0.123	0.534	0.495	0.821
Complexity:expert	0.115(0.083,0.170)	0.480 (0.366, 0.598)	0.505(0.431, 0.579)	0.779 (0.661, 0.858)
Complexity:non-expert	0.130 (0.112, 0.159)	0.588 (0.482, 0.682)	0.486 (0.390,0.583)	0.862(0.795, 0.894)

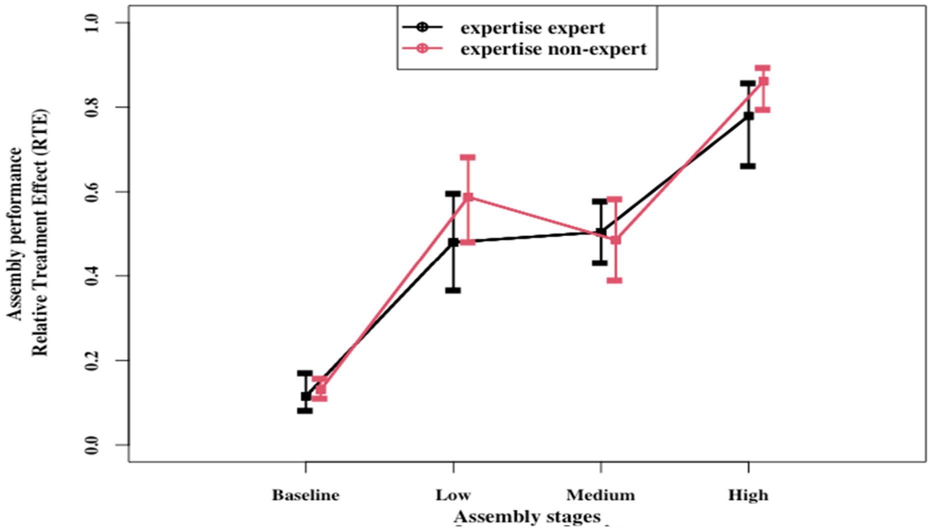

Figure S5. Relative treatment effects for assembly performance (completion time) within the interaction of task complexity and expertise.

Table S15. Multiple comparisons of parameter assembly performance in the engine assembly study with Bonferroni adjustment

**Comparison**	**Hypothesis**	**ANOVA-Type Statistic p-value**	**Adjusted p-value (Bonferroni adjustment)**
Baseline vs Low TC	Complexity:H0: $\mu_{baseline}=\mu_{low}$ Interaction:H0: $\mu_{experti} = \mu_{non\text{-}experti}$ for i = baseline, low.	p-value(complexity)<0.0001**** p-value(interaction) = 0.703	p-value(complexity)<0.0001**** p-value(interaction) =4.220
Baseline vs Medium TC	Complexity:H0: $\mu_{baseline}=\mu_{medium}$ Interaction:H0: $\mu_{experti} = \mu_{non\text{-}experti}$ for i = baseline,medium.	p-value(complexity)<0.0001**** p-value(interaction)= 0.420	p-value(complexity)<0.0001**** p-value(interaction)= 2.521
Baseline vs High TC	Complexity:H0: $\mu_{baseline}=\mu_{high}$ Interaction:H0: $\mu_{experti} = \mu_{non\text{-}experti}$ for i = baseline,high.	p-value(complexity)<0.0001**** p-value(interaction) = 0.487	p-value(complexity)<0.0001**** p-value(interaction)= 2.920
Low TC vs Medium TC	Complexity:H0: $\mu_{low}=\mu_{medium}$ Interaction:H0: $\mu_{experti} = \mu_{non\text{-}experti}$ for i =low,medium.	p-value(complexity)= 0.486 p-value(interaction)= 0.098	p-value(complexity)= 2.914 p-value(interaction)= 0.587
Low TC vs High TC	Complexity:H0: $\mu_{low}=\mu_{high}$ Interaction:H0: $\mu_{experti} = \mu_{non\text{-}experti}$ for i =low,high.	p-value(complexity)<0.0001**** p-value(interaction)= 0.870	p-value(complexity)<0.0001**** p-value(interaction)= 5.217
Medium vs High TC	complexity: H0: $\mu_{medium}=\mu_{high}$ Interaction:H0: $\mu_{experti} = \mu_{non\text{-}experti}$ for i =medium,high.	p-value(complexity)<0.0001**** p-value(interaction)= 0.385	p-value(complexity)<0.0001**** p-value(interaction)= 2.307
